# Accumulation of Kv7.2 channels in putative ectopic transduction zones of mice nerve-end neuromas

**DOI:** 10.1186/1744-8069-7-58

**Published:** 2011-08-14

**Authors:** Carolina Roza, Sol Castillejo, Jose A Lopez-García

**Affiliations:** 1 Dpto. Fisiología. Edificio de Medicina Universidad de Alcalá. Alcalá de Henares, 28871 Madrid, Spain

## Abstract

**Background:**

Modulation of M-type currents has been proposed as a new strategy for the treatment of neuropathic pain due to their role in regulating neuronal excitability. Using electrophysiological techniques we showed previously that the opening of Kv7 channels with retigabine, blocked ectopic discharges from axotomized fibers but did not alter transduction at intact skin afferents. We hypothesized that after nerve damage, accumulation of Kv7 channels in afferent fibers may increase M-type currents which then acquired a more important role at regulating fiber excitability.

**Findings:**

In this study, we used an immunohistochemical approach to examine patterns of expression of Kv7.2 channels in afferent fibers after axotomy and compared them to patterns of expression of voltage gated Na^+ ^channels (Nav) which are key electrogenic elements in peripheral axons known to accumulate in experimental and human neuromas.

Axotomy induced an enlargement and narrowing of the nodes of Ranvier at the proximal end of the neuroma together with a dramatic demyelination and loss of structure at its distal end in which naked accumulations of Nav were present. In addition, axotomy also induced accumulations of Kv7.2 that co-localized with those of Nav channels.

**Conclusions:**

Whilst Nav channels are mandatory for initiation of action potentials, (i.e. responsible for the generation/propagation of ectopic discharges) an increased accumulation of Kv7.2 channels after axotomy may represent a homeostatic compensation to over excitability in axotomized fibers, opening a window for a peripheral action of M-current modulators under conditions of neuropathy.

## Background

Recent data from our laboratory showed that the specific Kv7 channel opener, retigabine, blocked ectopic discharges recorded from single axotomized fibers in response to stimulation of the neuroma [1]. This action was not modality specific but rather dependent upon hyperpolarization. However, modulation of M-currents did not change responses to stimulation of intact skin receptors. On the basis of these experimental observations we proposed that the prominent role of M-type currents newly acquired after axotomy emerged as a consequence of the accumulation of Kv7 channels in the vicinity of newly formed transduction zones known to occur at neuromatose endings.

Nerve damage has been shown to produce local demyelination and disruption of the molecular organization of nodes [2] characterized by large accumulations of voltage gated Na^+ ^channels (Nav) which are essential for the initiation of action potentials [3-6]. M-type currents have an important role stabilizing membrane potential at rest [7] and preliminary data suggest that ion channel rearrangement at ectopic areas could include an altered expression of Kv7 subunits [8].

Several lines of evidence suggest the presence of Kv7 channels/M-type currents at specialized receptor endings [9,10] and at nodes of Ranvier co-localizing with Nav channels [11,12]. Although there is no data on the distribution of Kv7 channels along unmyelinated axons, they are present in small diameter dorsal root ganglion neurons [13,14]. Kv7.2 is the subunit predominantly expressed in nodes of Ranvier [11,12,15] and may form homomeric channels to yield particular M-type currents [12].

Here we have examined Kv7.2 channel reorganization at aberrant transduction zones produced by saphenous nerve sectioning to further understand the enhanced role of M-type currents after axotomy. To this end, we have used Nav channel labeling as a marker of nodes and putative transduction zones formed after axotomy.

## Findings and discussion

The antibodies used in this study as well as the dilution conditions, are specified in table 1. Nodes were marked by the expression of Nav channels using a Pan Nav antibody. In order to study the expression of Kv7.2 we used five different antibodies which yielded similar results. PanNav and Kv7.2 immunofluorescence at nodes of Ranvier was eliminated by preincubation of each of the antibodies with the same concentration of the control antigen peptide (data not shown). Additional markers were used for myelin (Myelin P2), paranodal (CASPR) and juxtaparanodal areas (Pan NF).

**Table 1:** Antibodies, dilutions and sources

Antibody	Immunogen	Manufacturer, species antibody was raised in, mono- vs. Polyclonal	Dilution used
Pan Nav	1500-1518 aa of the α-subunit of Voltage Gated Sodium Channels	Chemicon, Rabbit, polyclonal	1:800

Myelin P2	The epitope for sc-49303 maps within amino acids 50-100 in the internal region of Myelin P2 of human origin (accession #P02689)	Santa Cruz, Goat, Polyclonal	1:100

CASPR	Fusion protein 1308-1381 (cytoplasmic domain) of rat Caspr (accession number #P97846)	NeuroMab (UC Davis, Davis CA), mouse monoclonal, clone K65/35	1:500

Pan NF	Fusion protein 1066-1174 (intracellular domain common to NF -155 and NF-186) of rat NF-155 (accession number AAL27854)	NeuroMab (UC Davis, Davis CA), mouse monoclonal, clone L11A/41	1:500

Kv7.2	aa 578-593 at the C-terminal domain	Alomone, rabbit, polyclonal	1:200

Kv7.2	aa 578-593 at the C-terminal domain	Chemicon, rabbit, polyclonal	1:400

Kv7.2	aa 578-593 at the C-terminal domain	Sigma, rabbit, polyclonal	1:200

Kv7.2	C-18; The epitope for sc-7792 maps within the last 50 amino acids at the C-terminus of KCNQ2 of human origin (accesion #O43526)	Santa Cruz, goat, polyclonal	1:800

Kv7.2	N-19; The epitope for sc-7793 maps within the first 50 amino acids at the N-terminus of KCNQ2 of human origin (accession #O43526)	Santa Cruz, goat, polyclonal	1:1000

**Figure 1:**
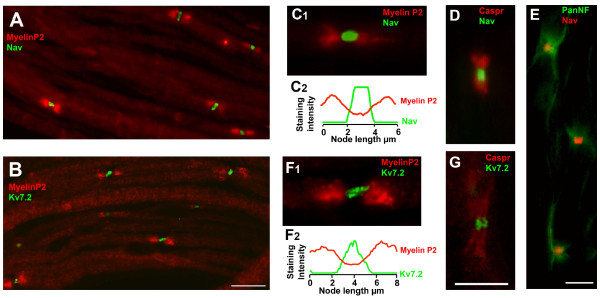
**Expression of Nav and Kv7.2 channels in nodes of Ranvier of intact fibres. *A ***and ***B ***show teased fibers form normal saphenous nerves immunostained for Myelin P2 and Pan Nav (*A*) or Kv7.2 (*B*). In this and following figures, color codes for antibodies are specified on images. Note that the fibers are well structured and nodes are easily identifiable. The following figures show high magnification images of nodal Nav channels flanked by depositions of Myelin P2 (***C_1_***), Caspr (***D***), Pan NF (***E***). ***C_2 _***shows the profile of immunofluorescence intensity along the nodal/paranodal zone shown in *C_1 _*(in this and following figures intensity curves are given in arbitrary units). ***F ***and ***G ***show nodal zones costained for Kv7.2 and Myelin P2 (***F_1_***) or Caspr (***G***). ***F_2 _***shows the profile of immunofluorescence intensity along the nodal/paranodal zone shown in *F_1_*. Scale bars: for *A *and *B*, 20 μm (shown in *B*); for *C_1_, D, F_1 _*and *G*, 5 μm (shown in *G*); for *E*, 5 μm.

Using our protocols and selection of antibodies, it was possible to identify and follow single fibers in our tissue samples of normal nerves (see Figure 1). Accumulations of Nav and Kv7.2 were flanked by depositions of any of the other markers at nodes of Ranvier. In between nodes, prolonged extensions of myelin were found. The staining patterns for all antisera were similar to what has been described in previous reports by other groups [2,5,11,12,16,17].

**Figure 2:**
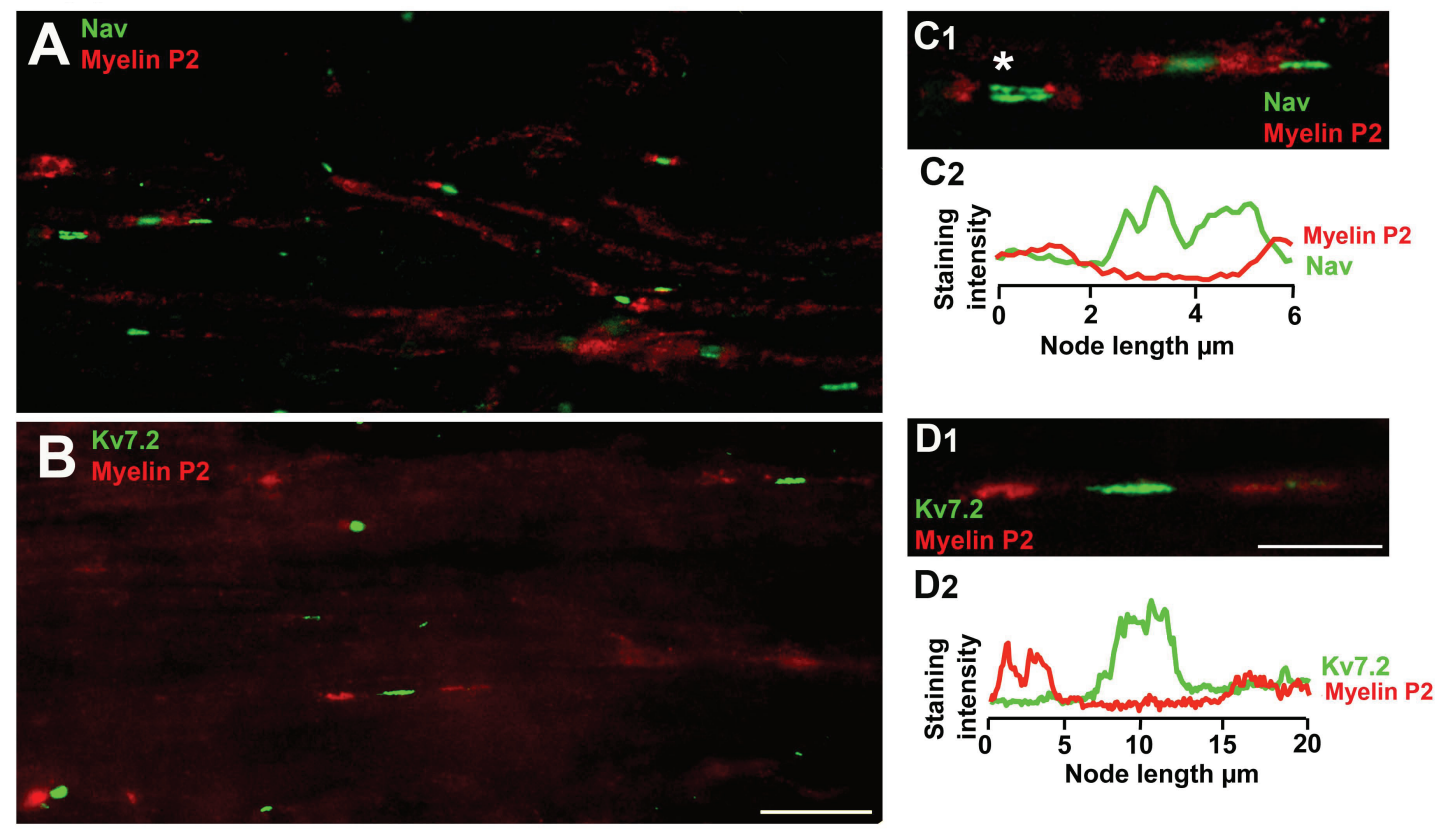
**Expression of Nav and Kv7.2 channels in nodes of proximal neuromas. *A ***and ***B ***show isolated teased fibers from nerve-end neuromas immunostained for Myelin P2 and Pan Nav (*A*) or Kv7.2 (*B*). In this proximal zone of the neuroma, the structure of the fibers was conserved and individual nodes/paranodes were still identifiable. ***C_1 _***shows a high magnification image of three nodal zones stained with Pan Nav and Myelin P2; ***C_2 _***shows the immunostaining intensity profile corresponding to the nodal zone marked with an asterisk in *C_1_*. ***D_1 _***shows a high magnification image of a nodal zone stained for Kv7.2 and myelin with its corresponding profile of immunostaining intensity (***D_2_***). Scale bars: for *A *and *B*, 10 μm (shown in *B*); for *C_1 _*and *D_1_*, 5 μm (shown in *D*).

**Figure 3:**
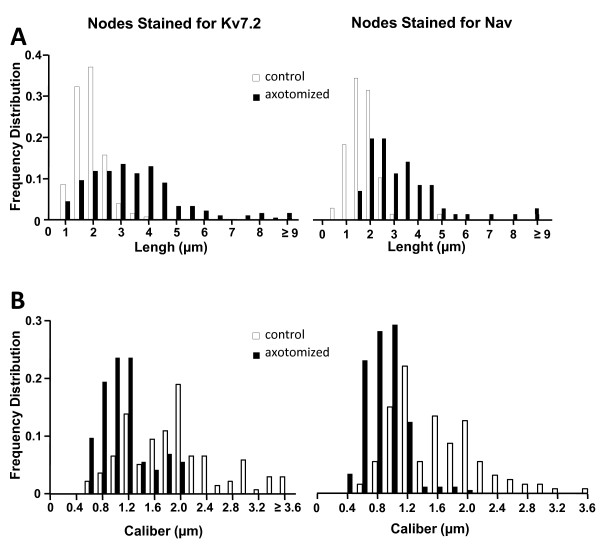
**Comparison of nodal parameters in normal vs. neuromatose fibres**. ***A***and ***B ***show the frequency distribution histograms for node length (*A*) and width (*B*). Kolmogorov-Smirnov test (*p *< 0.05 for all cases), indicates a non-Gaussian distribution in all cases.

The extent of axotomy-induced alterations in nodal structure was analyzed in the proximal end of the neuroma which preserved a certain structure (i.e. nodes and paranodes were still visible) (Figure 2). We measured the length and width of Nav and Kv7.2 aggregates in between myelin domains using double immunolabeling (Nav-Myelin P2 and Kv7.2-Myelin P2) from, at least, 7 slides obtained from a minimum of 6 different experiments. The mean values for each of these parameters are shown in Table 2. Frequency distribution plots for node length and width in intact nerves were similar for Nav and Kv7.2 (Figure 3). Axotomized fibers showed a significant reduction in the node caliber (1.7 and 1.8-fold for Nav and Kv7.2 stained fibers respectively) as well as a significant enlargement of nodal length (1.9 and 1.8-fold for Nav and Kv7.2 stained fibers respectively) as compared to intact fibers (see Table 2 and Figure 3).

**Table 2:** Measurements on nodes of Ranvier

	Nav		Kv7.2	
	
	Control	Axotomized	Control	Axotomized
Node Caliber (μm)	1.9 ± 0.06	1.1 ± 0.05*	1.5 ± 0.05 #	0.8 ± 0.02*
Node Length (μm)	1.7 ± 0.05	3.3 ± 0.2 *	1.9 ± 0.05 #	3.4 ± 0.13*

Values are given as Mean ± SEM. * Statistical difference between control and axotomized fibers stained for Nav or Kv7.2. # Statistical difference between fibers labeled for Nav and Kv7.2 (for both *p *< 0.05, Mann-Whitney test).

**Figure 4:**
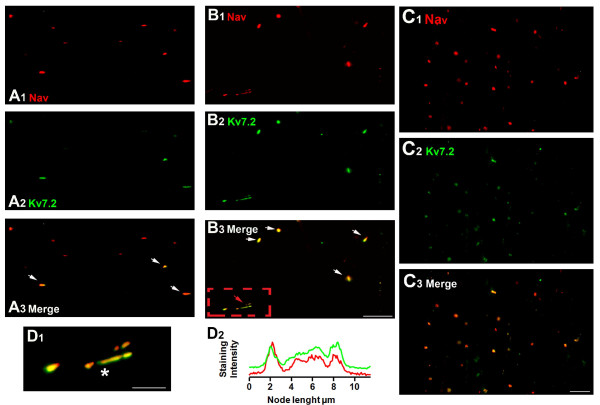
**Increase in Nav and Kv7.2 co-expression after axotomy. *A ***shows low magnification fields containing nodal accumulations of Nav (***A_1_***) and Kv7.2 (***A_2_***) channels in control nerve. ***A_3 _***shows a merge of A1 and A2 showing co-staining of some nodes (marked by arrows). ***B ***and ***C ***follow the same scheme as *A *but fields were obtained from the proximal end (*B*) or the distal end of the neuroma (*C*). Note the large proportion of co-staining in neuromatose fibers. ***D_1 _***shows a larger scale picture of a long nodal formation which corresponds to the area in the red square of *B_3_*. ***D_2 _***shows the immunostaining intensity profile corresponding to the nodal zone marked with an asterisk in *D_1_*. Scale bars: for *A *and *B *10 μm (shown in *B_3_*); for *C*, 10 μm (shown in *C_3_*); for *D_2_*, 5 μm.

Double labeling experiments were designed to establish the percentage of coincidence between Nav and Kv7.2 staining. Counts were made in 7 slides from 6 different experiments. In intact fibers a total of 258 nodes showed Nav channel accumulation and, of those, only 124 co-stained for Kv7.2 channel yielding a 48% of co-staining. The co-staining was markedly increased in neuromatose fibers, in which the proportion of coincidence raised significantly up to an 84% (out of 303 nodes marked for Nav, 254 co-stained for Kv7.2; Figure 4A and 4B, *p *< 0.001, Fisher exact test). A high coincidence of co-staining was also visible on the neuromatose areas closer to the injury site (Figure 4C).

**Figure 5:**
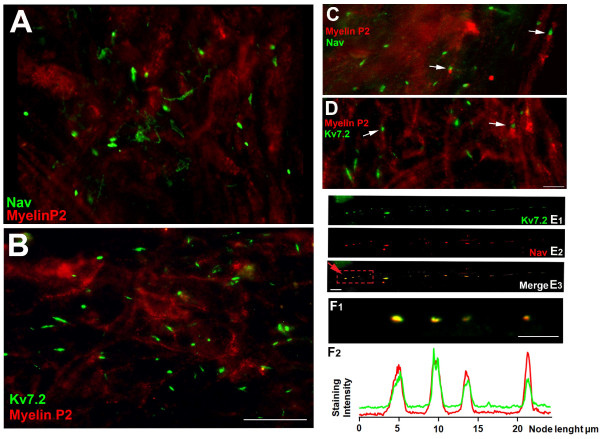
**Abnormal expression of Nav and Kv7.2 channels in distal neuromas. *A ***and ***B ***are medium scale magnification fields obtained from distal areas of the neuroma. In both instances there is a clear lack of structure and myelin loss. In addition, large accumulations of Nav and Kv7.2 channels are shown in *A *and *B *respectively. ***C ***and ***D ***contain field images of transition areas in which normal nodes (marked by arrows) are detected close to larger accumulations of Nav (*C*) or Kv7.2 (*D*). ***E ***shows the co-staining of Nav and Kv7.2 in a dotted line-like structure, probably an indication of a demyelination/remyelination process within the same fiber. ***F_1 _***shows several individual nodes at a high magnification (contained in red square in *E_3_*). The corresponding immnofluoresce intensity profiles are shown in ***F_2_***. Scale bars: for *A *and *B*, 20 μm (shown in *B)*; *C *and *D*, 5 μm (shown in *D*); for *E*, 5 μm (shown in *E_3_*) and for *F_1_*, 5 μm.

At the most distal part of the neuroma, the general organization of the nerve was completely lost and it was not possible to trace the path of individual fibers (see Figure 5A and 5B). Normal nodal-like structures were sparse (Figure 5C and 5D) and dense naked accumulations of Nav or Kv7.2 channels were clearly seen (see Figure 5A and 5B). Occasionally, intermittent dispositions of node-like formations at short internodal intervals were also observed (Figure 5E).

The data presented here confirms previous observations on the disruption of axonal organization following axotomy [2,6]. The original contribution of the present work is the confirmation of an abundant expression of Kv7.2 channels in newly formed aberrant loci, which also express abundant Nav channels and presumably represent newly transduction zones [4,18,19].

At the distal end of the neuroma, we observed severe loss of myelin and nerve organization where the development of naked accumulations of Nav and Kv7.2 becomes a salient feature. In the proximal end of the neuroma, we observed a significant two-fold widening of the nodes together with a decreased node caliber which still expressed Nav or Kv7.2 channels in a continuous form. Large increases of nodal length have been reported for tissue samples obtained from multiple sclerosis patients [20]. Bostock and Sears [21] reported continuous conduction from chemically demyelinited rat spinal roots, demonstrating that bared internodal axons can become excitable. This feature is likely due to the presence of Nav channels at the site of injury and also in newly formed nodes of Ranvier during remyelination [6]. Furthermore, Nav channel accumulation has been also described in fibers from human painful neuromas [22] and painful human dental pulp [19]. A recent study demonstrated that 1/3 of the patients that underwent surgical repair after peripheral nerve section and still suffer from chronic pain, exhibited a significant slowing in the conduction velocities of their repaired nerves as compared to pain-free patients, probably due to less successful nerve regeneration [23]. These series of events reinforce the connection of Nav channel clusters with ectopic loci and pain.

Schwarz [12] reported that Kv7.2 was present in nodes of fibers from all diameters, and, although no co-staining with Nav was performed, the staining pattern of both Kv7.2 and Nav were similar. Our present results confirm these previous observations and, in addition, show that following axotomy, the occurrence of co-localization of Nav and Kv7.2 channels increase considerably (1.75-fold increase) in proximal areas of the neuroma where nerve structure and myelin are relatively conserved. Nav and Kv7.2 channels share an ankyrin-G domain for membrane retention [11], and elevated levels of ankiryn-G have been reported in samples from human painful neuromas [24]. This is supposed to facilitate Nav channel insertion into the axon membrane [25] and may facilitate the insertion of Kv7.2 channels as well.

## Conclusions

The present immunohistochenical data, indicate the presence of Kv7.2 channels in a proportion of nodes of the intact nerve as reported by others [11,12,15], although M-type currents seem to have only a minor role at coding acute nociceptive stimuli of cutaneous origin [1]. After axotomy, there is an accumulation of Kv7.2 channels which parallels that of Nav channels in de-myelinated areas thought to constitute ectopic loci [3,19]. A concentration of Kv7.2 channels is likely to result in larger M-like currents which then acquire a more preponderant role at controlling axonal excitability. These results open up the possibility of interacting with peripheral Kv7.2 channels to attenuate neuropathic pain symptoms.

## Methods

Adult outbreed CD1 mice of both sexes were used. European Union and State legislation for the regulation of animal experiments were followed and the local Animal Care Facility approved the experimental protocols. Nerve-end neuromas were produced by total section of both saphenous nerves in mice following the methods previously described [1]. Under deep anesthesia with isofluorane (~3.5-4% in pure O2) and with sterile precautions, the saphenous nerve was exposed at the level of the mid-thigh, dissected free and tightly ligated with 8-0 silk. The nerve was cut distal to the ligature and the cut end inserted into a 3 mm long silicone tube (0.45 mm internal diameter) to prevent lateral innervation of surrounding tissue. The tube was tied in place with the same piece of silk and a ~2 mm piece of the distal nerve stump was excised to prevent reinnervation. The incision was closed. The animals were housed in groups of two to four and inspected periodically for infections or abnormal behavior. The mice had access to water and food *ad libitum*. Saphenous nerves from naive mice were used as controls.

Animals were perfused with cold PBS and either intact nerves or ~4 week old nerve end-neuromas were extracted, embebed in OCT (Tissue-Tek) and submerged in acetone previously cooled in dry ice for a minimum of 30 minutes. Preparations were placed in cold PBS solution and fibres were teased out from samples, transferred to Superfrost poly-L-lysine coated slides (SuperFrost-Plus, Menzel-Glaser) and allowed to dry. In each slide, teased samples from normal nerves and nerve-end neuromas were present to minimize potential inter-staining variability. Slides were stored at -20°C until used for staining with antibodies.

Slides were permeabilized by immersion in -20°C acetone for 10 minutes. After washing with PBS, non-specific binding sites were blocked for 1 hour at room temperature (TBS containing 5% gelatin fish, 0.5% Triton X-100). Slides were incubated overnight at 4°C in a humidified chamber with primary antibodies (see Table 1) in blocking buffer (0.2% of Triton X-100). Slides were then washed and incubated for 2 hours at room temperature with the appropriate secondary antibodies (cyanine-2-conjugated and cyanine-5-conjugated diluted 1:800 and 1:200, respectively, Jackson Immunoresearch Labs) in blocking buffer. After washing, slides were cover-slipped using Fluor-Save antifade reagent. These methods have been adapted from previous relevant publications in the literature [11,12].

Tissue specimens were evaluated with an Olympus Fluorescence Microscope. Olympus software Cell-R was used for acquisition of images. Final image processing for illustration purposes (i.e. scales, improve in brightness and framing) was done with Adobe Photoshop.

## Competing interests

The authors declare that they have no competing interests.

## Authors' contributions

SC carried out immunohistochemistry and participated in data collection. CR and JAL carried out conception and design, analysis and interpretation of data, drafting, critical revising and final approval of the manuscript. All authors read and approved the final manuscript.
